# Arresting chromosome replication upon energy starvation in *Escherichia coli*

**DOI:** 10.1007/s00294-021-01202-2

**Published:** 2021-08-03

**Authors:** Godefroid Charbon, Jakob Frimodt-Møller, Anders Løbner-Olesen

**Affiliations:** Department of Biology, University of Copenhagen, Ole Maløes Vej 5, 2200 Copenhagen N, Denmark

**Keywords:** Cellular Energy Status, Chromosome replication, DnaA, Genome stability

## Abstract

Most organisms possess several cell cycle checkpoints to preserve genome stability in periods of stress. Upon starvation, the absence of chromosomal duplication in the bacterium *Escherichia coli* is ensured by holding off commencement of replication. During normal growth, accumulation of the initiator protein DnaA along with cell cycle changes in its activity, ensure that DNA replication starts only once per cell cycle. Upon nutrient starvation, the prevailing model is that an arrest in DnaA protein synthesis is responsible for the absence of initiation. Recent indications now suggest that DnaA degradation may also play a role. Here we comment on the implications of this potential new layer of regulation.

## Introduction

Nutrient availability affects the cell cycle of all organisms. In Eukaryotes, checkpoints known as ‘Restriction’ or ‘Start’ prolong the G1 phase following nutritional stress until a favorable cellular energy state is reached (Johnson and Skotheim 2013; Solaki and Ewald 2018). In the well-studied prokaryotic organism *Escherichia coli*, no such checkpoints have been identified. Instead, accumulation of the initiator protein DnaA, whose expression is coupled to mass increase, is thought to play a determining role in deciding when DNA synthesis starts (Lobner-Olesen et al. 1989; Si et al. 2017).

### Initiation of DNA replication depends on accumulation and activation of DnaA

**Fig. 1:**
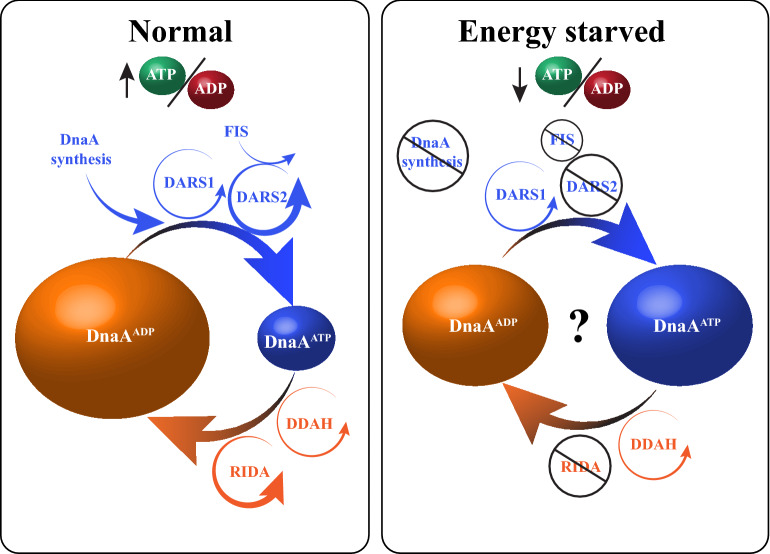
Regulation of DnaA activity. During normal growth (left), DnaA^ATP^ (blue) is formed through DnaA synthesis (apo-DnaA binding ATP that is more abundant than ADP) and by DARS ‘rejuvenation’ (*DARS1* and FIS-dependent *DARS2*) through dissociation of ADP from DnaA^ADP^ (orange). The formation of DnaA^ADP^ is controlled by DDAH and RIDA through stimulaion of DnaA ATPase activity. On average, total DnaA is constituted of ~ 30% DnaA^ATP^ and ~ 70% DnaA^ADP^ in exponentially growing cells. In energy starved cell (right), *DARS2* and RIDA are both inactive, and the ratio DnaA^ATP^/DnaA^ADP^ is not known

DnaA is a multi-domain protein that binds the origin of replication and loads the replicative helicase (Hansen and Atlung 2018; Katayama et al. 2017; Leonard and Grimwade 2015; Riber et al. 2016). Like other initiator proteins, it possess an AAA + domain with ATPase activity that determines its ability to form a nucleoprotein structure on the origin and to load the helicase; the initiator being active when bound to ATP while inactive in its ADP bound form. Because DnaA has an equally high affinity for ATP and ADP and because ATP is about seven times more abundant in the cell than ADP during normal growth, it is assumed that newly synthesized DnaA mainly binds ATP. In other words, the energy state of the cell could influence the ratio of active to inactive initiator. Thus it is expected that overproduction of DnaA will increase the amount of DnaA^ATP^ relative to DnaA^ADP^. Accordingly, overproduction of a mutant DnaA that binds ATP and ADP equally well but has lost its ATPase activity (DnaAR334A), results in an increase of DnaA^ATP^ from ~ 10% to ~ 60% and an enormous increase in initiation frequency (Nishida et al. 2002). Contrary to the expectation, overproduction of wild-type DnaA does not increase or even reduce the ratio DnaA^ATP^/DnaA^ADP^ (Kurokawa et al. 1999; Nishida et al. 2002) and has a relatively minor effect on the initiation frequency (Nishida et al. 2002; Riber et al. 2006). The fact that an accumulation of DnaA^ATP^ alone is much more potent in starting DNA replication than an accumulation of DnaA^ATP^ together with DnaA^ADP^ indicates that DnaA^ADP^ has an inhibitory function on initiation. Thus, DnaA needs to be both in sufficient amount and predominantly in active form (high DnaA^ATP^/DnaA^ADP^ ratio) to start DNA replication (Fig. 1). DnaA is predominantly found in the ADP bound form at the population level (~ 70%) (Kato and Katayama 2001). This is because the nucleotide bound state of DnaA is determined by a balance between control mechanisms.

### Regulation of DnaA activity

On one hand, there is Regulatory Inactivation of DnaA (RIDA) (Kato and Katayama 2001) and *datA*-dependent DnaAATP hydrolysis (DDAH) (Kasho and Katayama 2013) that stimulate DnaA ATPase activity to reduce the DnaA^ATP^/DnaA^ADP^ ratio. On the other hand, the rejuvenation of DnaA^ADP^ to DnaA^ATP^ at DnaA Rejuvenating Sequences *DARS1* and *DARS2* serves to increase the DnaA^ATP^/DnaA^ADP^ ratio by promoting the dissociation of ADP from DnaA (Fujimitsu et al. 2009); the Apo-DnaA produced binds preferentially ATP (Fig. 1). These regulatory mechanisms act in concert during the cell cycle to ensure that DnaA^ATP^ accumulates pre-initiation and is subsequently converted to DnaA^ADP^ post-initiation. Mechanistically, DDAH at the *datA* locus and rejuvenation at *DARS* sites distinguish themselves by the type of DnaA-DNA complex formed and the auxiliary proteins required to function. *DARS1* is constitutively active and does not require helper proteins while IHF is required for DDAH and *DARS2*. Rejuvenation at *DARS2* rejuvenation is also depends on Fis function and is expected to be growth phase regulated (Frimodt-Moller et al. 2016; Inoue et al. 2016; Kasho et al. 2014) because Fis is abundant in exponential phase but absent during stationary phase. RIDA, is orchestrated by the Hda protein interacting with the DNA loaded β-clamps. During replication, β-clamps tether the DNA polymerase to the template DNA. However, numerous β-clamps are left loaded on double stranded DNA behind the replication forks for several minutes (Moolman et al. 2014). These DNA-bound β-clamps act as a platform for several activities such as DNA repair and in the case of RIDA serve to complex DnaA with the protein HdA resulting in activation of DnaA ATPase. RIDA is expected to be less active in absence of DNA replication where few β-clamps are left loaded on DNA (Katayama et al. 1998; Kurokawa et al. 1999). Because RIDA is the predominant process lowering the DnaA^ATP^/DnaA^ADP^ ratio, we speculate that DnaA becomes more ATP-bound during stationary phase and energy starvation (Fig. 1). In cells where only DDAH and *DARS1* are functional, the DnaA^ATP^/DnaA^ADP^ ratio increases (Fujimitsu et al. 2009). Furthermore, DnaA^ADP^ is slowly converted into DnaA^ATP^ in a RIDA-deficient cell when protein synthesis is blocked by chloramphenicol treatment (Fujimitsu et al. 2009; Kurokawa et al. 1999).

### DnaA synthesis arrest upon starvation

**Fig. 2:**
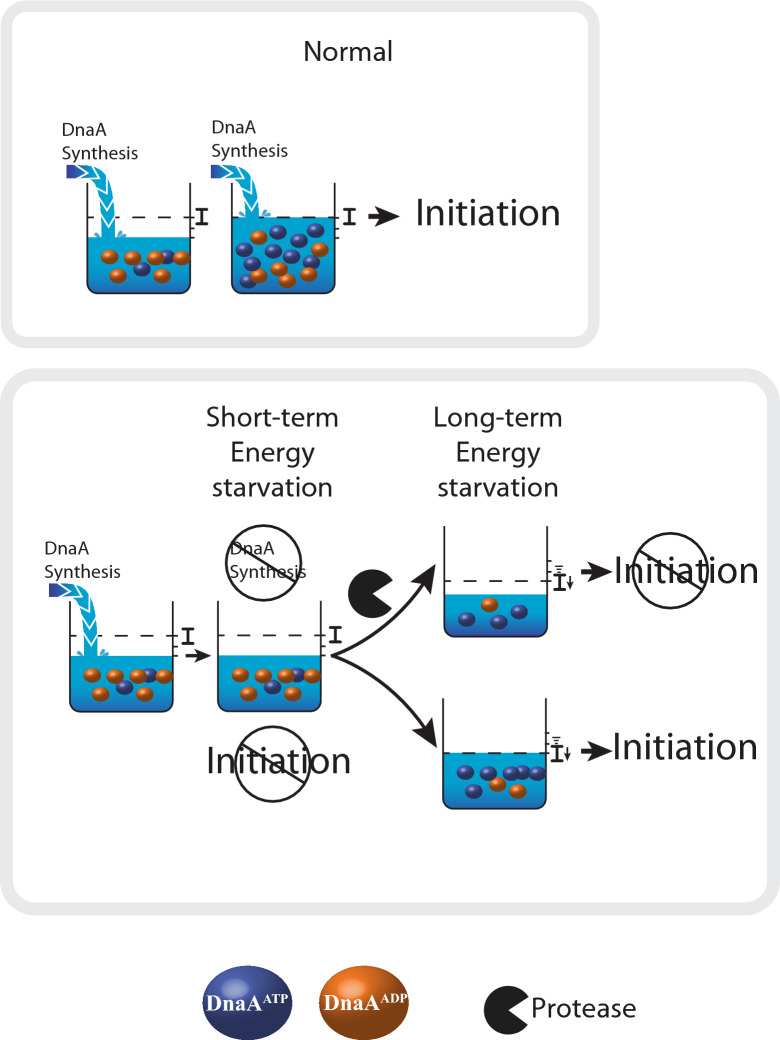
A role for degradation of DnaA during energy etarvation. During normal growth (upper panel), DNA replication is initiated when enough DnaA molecules have accumulated and when DnaA^ATP^/DnaA^ADP^ is high (level of DnaA required to for initiation: I). During short-term energy starvation (lower panel), DnaA does not accumulate to sufficient level and DnaA^ATP^/DnaA^ADP^ is low. During long-term energy starvation, we propose that DnaA^ATP^/DnaA^ADP^ becomes sufficiently high to allow for replication initiation despite of fewer DnaA molecules, unless DnaA level is lowered by degradation

Upon energy starvation, DNA replication stops and it was assumed that a general reduction in protein synthesis (Holm et al. 2010), including DnaA, is responsible for this arrest (Fig. 2). In this model, DnaA fails to accumulate to a level sufficient to promote a new round of replication. The DNA replication arrest is specific to the initiation step, with ongoing replication allowed to proceed to completion. This is also the case when DnaA expression is blocked by treatment with antibiotics that arrest protein synthesis such as chloramphenicol and rifampicin or during the stringent response when the alarmone (p)ppGpp is induced (Chiaramello and Zyskind 1990; Lark 1972; Schreiber et al. 1995; Skarstad et al. 1986). This is corroborated by the fact that the hyperactive DnaA mutant DnaAcos continues to initiate DNA replication in the presence of chloramphenicol (Kellenberger-Gujer et al. 1978) and by the observation that overexpression of *dnaA* bypasses the DNA replication arrest seen in (p)ppGpp-induced cells (Riber and Lobner-Olesen 2020).

### DnaA degradation during ATP starvation

Recently, a manipulation of the cellular ATP level indicates that additional regulation mechanism exist. Normally, DnaA is extremely stable during steady state growth, with protein half-life exceeding 24 h (Atlung and Hansen 1999; Torheim et al. 2000). It is shown now that depletion of ATP results in a replication initiation arrest as expected, but surprisingly, this is accompanied by degradation of about 30% of the DnaA proteins after 1-hour ATP starvation (Charbon et al. 2021). The nucleotide binding of DnaA does not affect DnaA stability during energy starvation (Charbon et al. 2021). However, the mechanism responsible for the degradation remains elusive and neither of the ATP-dependent proteases Lon or ClpP, implicated in the initiator proteolysis in the bacterium *Caulobacter crescentus*, are involved. Following carbon starvation, a similar DNA synthesis arrest is observed despite the fact that DnaA is stable during the initial stages of starvation where ATP/ADP level is remains unchanged. Nonetheless, following long-term carbon starvation, the ATP/ADP level falls (Chapman et al. 1971), and DnaA is degraded (Charbon et al. 2021).

### DnaA degradation during stringent response

Although DnaA was originally described as stable or even more abundant in stationary phase (Ali Azam et al. 1999; Sakakibara and Yuasa 1982; Sekimizu et al. 1988), recent work indicates that DnaA is degraded during the stringent response (increased levels of (p)ppGpp) occurring upon entry into stationary phase, or upon amino acid starvation (Gross and Konieczny 2020). Indeed, the amount of DnaA per cell decreases over time following induction of (p)ppGpp, a decrease that is not observed in a *lon* or *ppk* mutant (polyphosphate synthesis). In vitro, Lon specifically degrades DnaA^ADP^ in the presence of polyphosphate (PolyP) (Gross and Konieczny 2020). Thus it is proposed that accumulation of PolyP acts as a starvation signal to degrade DnaA and prevent initiation of DnaA replication. Note that the degradation of DnaA apparently depends on how (p)ppGpp is induced as it is not observed elsewhere (Riber and Lobner-Olesen 2020).

### A new regulatory mechanism?

Because *dnaA* expression is reduced or arrested during energy starvation and the stringent response respectively, future work should address the need for DnaA degradation as extra layer of regulation. For example, for cells entering carbon starvation, the DNA replication block does not require DnaA degradation (Charbon et al. 2021). In this case, cessation of de novo DnaA synthesis is sufficient to arrest initiation and degradation of DnaA comes only much later. The question therefore remains why DnaA degradation in ATP depleted or (p)ppGpp accumulating cell is at all necessary. One possibility is that DnaA degradation is incidental; i.e., DnaA instability may be a mere consequence of a protein degradation in general. This explains why mutants in proteolytic pathways have not been isolated in genetic screens for suppression of DnaA hyperactivity (Charbon et al. 2018). On the other hand, although the block in expression of DnaA is a sufficient short-term solution, we suggest that it becomes ineffective if the DnaA^ATP^/DnaA^ADP^ balance increases over time (Fig. 2). We speculate that upon long-term energy starvation during which RIDA and *DARS2* are inactive, DnaA^ADP^ is converted into DnaA^ATP^ overtime, thereby lowering the number of DnaA molecules needed to trigger initiation of replication (Fig. 2). Thus, DnaA is capable to re-initiate DNA replication despite being at a level normally deemed insufficient. This is in accordance with the observation that a hyperactive DnaA protein mimicking DnaA^ATP^ can continue to initiate new rounds of replication despite protein synthesis arrest (Kellenberger-Gujer et al. 1978). Degradation of DnaA in general during ATP depletion (Charbon et al. 2021) could lower the cellular amount of protein and prevent this (Fig. 2). The observation that specifically DnaA^ADP^ is degraded during PolyP accumulation (Gross and Konieczny 2020) is counterintuitive, as this would increase the DnaA^ATP^/DnaA^ADP^ ratio and theoretically lower the amount of DnaA needed to start replication; fewer DnaA molecules but more active. Since DnaA^ADP^ is the most abundant species, accounting for 70% of total DnaA, DnaA^ADP^ proteolysis could provide a fast way to degrade the majority of DnaA molecules. However this is not supported by the modest ~ 20% reduction in total DnaA observed after 30-min amino acid starvation (Gross and Konieczny 2020). On the other hand, degrading the inactive form of DnaA could be a way to quickly restart DNA replication following starvation. Post starvation, cells would be left with only DnaA^ATP^ and therefore fewer DnaA molecules would be required to re-initiate. Finally, we do not exclude that the role of DnaA degradation is to control transcriptional activity (Messer and Weigel 1997) instead of or in addition to its initiator activity. Indeed, DnaA overproduction during late phase has been reported to induce the transcription of *polA* gene encoding DNA polymerase I in a manner independent of the late phase sigma factor RpoS (Quinones, et al. 1997). Thus, DnaA transcriptionial activity could clash with other master regulators specialized in promoting survival during starvation, justifying DnaA degradation.

## Data Availability

Not relevant.

## Code availability

Not relevant.
